# A retrospective study of end-stage kidney disease patients on maintenance hemodialysis with renal osteodystrophy-associated fragility fractures

**DOI:** 10.1186/s12882-020-02224-7

**Published:** 2021-01-11

**Authors:** Lihua Xie, Xuantao Hu, Wenzhao Li, Zhengxiao Ouyang

**Affiliations:** 1 Department of Nephrology, The Second Xiangya Hospital, Central South University, Changsha, 410011 Hunan P.R. China; 2 Department of Orthopedics, The Second Xiangya Hospital, Central South University, 139 Renmin Road, Changsha, 410011 Hunan P.R. China

**Keywords:** ESRD, Fragility fracture, Risk factors, Prognosis

## Abstract

**Background:**

Nephropathy associated metabolic disorder induces high incidence of fragility fracture in end-stage renal disease (ESRD) patients. As the risk factors and prognosis of fragility fracture in ESRD patients are unclear, more research is needed. This study aimed to evaluate various risk factors for ESRD-related fragility fractures, explore factors affecting the prognosis of patients with such fractures, and provide information for prevention and treatment of renal osteopathy to improve the prognosis of patients.

**Methods:**

In this retrospective case-control study, the case notes of 521 ESRD patients who received maintenance dialysis for at least 3 months were examined. Finally, 44 patients diagnosed with fragility fractures were assigned to the fragility fracture (FF) group and 192 patients were included in the control group (CG). Demographic information, underlying diseases, nutritional, bone metabolism, and renal function parameters, along with the number and causes of any deaths, were recorded for multiple statistical analysis.

**Results:**

The FF group had increased incidences of essential hypertension and diabetes mellitus and higher serum calcium, corrected calcium, alkaline phosphatase, and hemoglobin levels. Immunoreactive parathyroid hormone (iPTH), total cholesterol (TC), and low density lipoprotein (LDL) levels were higher in the CG. Multivariate Cox regression analysis revealed that fragility fracture was an independent risk factor for all-cause mortality in ESRD patients (*P* < .001, RR: 4.877, 95% CI: 2.367–10.013).

**Conclusions:**

Essential hypertension and diabetes, high serum calcium and alkaline phosphatase levels, and reduced iPTH levels were risk factors for fragility fracture in ESRD patients. Maintaining iPTH and serum TC levels may protect against fragility fractures in them. Fragility fractures may yield poor prognosis and shorter lifespan. The presence of fragility fracture was an independent predictor of all-cause death in ESRD patients.

**Supplementary Information:**

The online version contains supplementary material available at 10.1186/s12882-020-02224-7.

## Background

End-stage renal disease (ESRD) is a crucial issue that affects a substantial number of people in developing countries. As the end-stage of chronic kidney disease (CKD), the prevalence of ESRD steadily increases along with the numbers of the elderly in a population and the ubiquity of some chronic diseases such as diabetes mellitus and hypertension. In ESRD patients, renal osteodystrophy is a common complication which can lead to abnormalities of calcium and phosphorus metabolism, and bone formation and turnover dysregulation [1]. Patients with ESRD therefore may develop symptoms, including bone pain, bone deformation, osteoporosis and even spontaneous fractures, which affect their physical and mental health, reduce their quality of life and even affects their prognosis.

In recent years, the predisposition to fractures in ESRD patients has not been given the attention it deserves and the annual incidence of ESRD-related fragility fractures becomes a serious problem, especially in developing countries [2, 3]. Kidney Disease Improving Global Outcome (KDIGO) has defined Chronic Kidney Disease and Mineral Bone Disease (CKD-MBD) as a clinical syndrome encompassing mineral, bone, and calcific cardiovascular abnormalities that develop in patients with CKD. They have also recommended the desirable serum content of calcium, phosphorus, and intact iPTH [4]. Despite these measures, there are still many shortcomings to be addressed. One of these is that most of the data and cases in existing research and treatment guidelines focus on western populations. Whether these data are suitable for specific ethnic and demographic situations in Asia remains to be seen. Furthermore, there have been relatively few reports in recent years on the clinical characteristics and prognosis of fragility fracture in ESRD patients. Thus, the aim of this retrospective study was to evaluate the various risk factors of ESRD-related fragility fractures, explore the factors affecting the prognosis of these patients, and provide information for clinical prevention and treatment of renal osteopathy to improve the prognosis of patients.

## Methods

We retrospectively analyzed all patients included in the clinic data bank in The Second Xiangya Hospital of Central South University which launched in 2000 and based on the medical history, biochemical and imaging results. The study protocol was approved by the institutional review board of Central South University. All the patients had accepted maintenance hemodialysis for at least 3 months. We examined the clinical information of all 521 patients from January 1st, 2013 to August 31st, 2019 as a research group in the data bank. Among these patients, all 44 cases of diagnosed fragility fracture were included in the FF group there were 20 males and 24 females. The 44 cases were hospitalized for bone pain that occurred spontaneously or sustained from a minor trauma and confirmed initial fragility fracture according to history of present illness and X-ray or CT examination. For patients with any signs (local pain, abnormal motivation, etc.) of bone fracture, X-ray were performed routinely. For fractures which remains elusive in local X-ray images, high-resolution CT and reconstruction were deployed. Radiological findings were analyzed by two observers blinded to clinical data, with an interobserver concordance of 95%. A total of 192 hemodialysis patients without fracture hospitalized in the same period were selected as CG, in which, 100 of them were male. The Exclusion criteria included: (1) incomplete clinic information. (2) long-term or current use of glucocorticoids or selective estrogen receptor modulators. (3) severe infection or surgical trauma within 1 month. (4) presence of diseases that seriously affect bone metabolism, such as tumors, tuberculosis, Cushing’s syndrome, etc. (5) fractures caused by an external force, such as a car accident, etc. The nephropathy of these patients included: chronic glomerulonephritis (99 cases), diabetic nephropathy (31 cases), hypertensive nephropathy (27 cases), diabetes associated with hypertension nephropathy (biopsy proven combined diabetic nephropathy and hypertensive nephrosclerosis) (14 cases), polycystic kidney (autosomal dominant) (13 cases), chronic interstitial nephritis (10 cases), gouty nephropathy (10 cases), ischemic nephropathy (7 cases), obstructive nephropathy (8 cases), purpuric nephritis (3 cases) and unknown etiology nephropathy (14 cases). All patients were treated with conventional therapy including (1) phosphate binder, active vitamin D, and sensitive calcium receptor agonist (adjusting calcium and phosphorus metabolic disorders); (2) calcium channel blockers (CCBs), alpha-receptor blockers, beta receptor blockers, angiotensin-converting enzyme inhibitors (ACEIs), and angiotensin receptor blockers (ARB) (adjusting blood pressure); (3) insulin and oral medications (adjusting blood glucose); (4) recombinant human erythropoietin (rh EPO), iron preparations, and folic acid (curing the anemia); and (5) hemodialysis (2–3 times per week), etc.

### Experimental cases and data collection

1. Baseline demographic data (age, gender) and clinical data related to chronic nephropathy (the history of dialysis, adequacy of dialysis(kt/v) and underlying disease such as hypertension and/or diabetes) were collected at the time of enrollment.
2. Both groups of patients had venous blood sample collection in the early morning before intended hemodialysis therapy to collect laboratory biochemical data, such as hemoglobin, albumin, alkaline phosphatase, serum calcium, serum phosphorus, serum lipid (TC – total cholesterol, TG - triglycerides, low-density lipoprotein, high-density lipoprotein, Lipoprotein α), iPTH. We calculated the calcium-phosphorus product and the correction for calcium. When serum albumin levels were lower than 40 g/L, the following formula [5] can be used: Z=X+ 0.2×[4-Y]. (Z: correction for calcium (mmol/l); X: serum calcium (mmol/l); Y: serum albumin (g/dl)). The fractures were assessed by X-ray or CT scan. We defined the fracture as a break in the bone’s continuity and integrity. Epiphyseal separation was also included in the definition.
3. The mean follow-up time for all patients was 1439.0 (745.0–2513.0) days. During the follow-up period, survival time, all-cause mortality and cardiovascular mortality was monitored.

### Statistical approach

SPSS 22.0 was used for statistical analysis, and all data were tested by the Kolmogorov-Smirnoff method for normal distribution. Mean ± standard deviation was used for recording measurement data conforming to normal distribution, and quartile was used for recording measurement data not conforming to normal distribution. The T-test or rank-sum test was used for analyzing differences between two groups of continuous variables, and categorical variables were expressed as percentage and analyzed by chi-square test. Logistic regression was used for multivariate analysis and calculating the relative risk ratio. The Kaplan-Meier survival analysis and the Log Rank test were used to estimate survival rate of all-cause mortality, cardiovascular event mortality and analyze the statistical significance of the survival rate differences between two groups. Cox regression analysis was used to study the effects of multiple factors on the survival period. *P* < .05 was considered statistically significant.

## Results

### Fracture in patients with ESRD

Our study found that among 521 ESRD patients, 44 suffered fragility fractures during the follow-up period, with a rate of 8.4% and an annual average incidence of about 2.76‰. The types of fracture included 13 cases of vertebral fractures (7 thoracic, 4 lumbar, and 2 thoracolumbar), 15 cases of proximal femoral fractures, 4 cases of radial fracture, 3 cases each of ossa pubis, fibula and proximal humerus fracture, 2 cases of pelvic fracture, and 1 case of scapula fracture. The most common fracture sites are the vertebral body and the proximal femur.

### Etiology analysis in ESRD patients with fragility fractures

The baseline information of patients in both groups is shown in Table 1. The average age of the FF group was 67.2 (range 57.0–78.0), the average age of the CG was 65.3 (range 56.0–79.0) There were no statistical differences in age, gender, adequacy of dialysis (kt/v), or the period of dialysis therapy between the fragility group and CG. The prevalence of hypertension and diabetes in the FF was, however, significantly higher than in the CG, (*P* < .05).

**Table 1:** Baseline information of both groups

	Fragility group (*n*=44)	Control group (*n*=192)	P
Age (years)	67.2 (57.0,78.0)	65.3 (56.0,79.0)	NS
Male/female ratio	20/24	100/92	NS
Primary hypertension (%)	24 (54.5%)	65 (33.9%)	0.022
Diabetes (%)	20 (45.5%)	54 (28.1%)	0.028
Period of dialysis therapy (months)	36.0 (12.0,72.0)	24.0 (16.0,48.0)	NS
Adequacy of dialysis (kt/v)	1.56 (1.37,1.65)	1.49 (1.32,1.61)	NS

*NS* None of significance; *n* Number

### Statistical analysis of the serum nutrition-related parameters and the mineral-bone metabolism disorder parameters

**Fig. 1:**
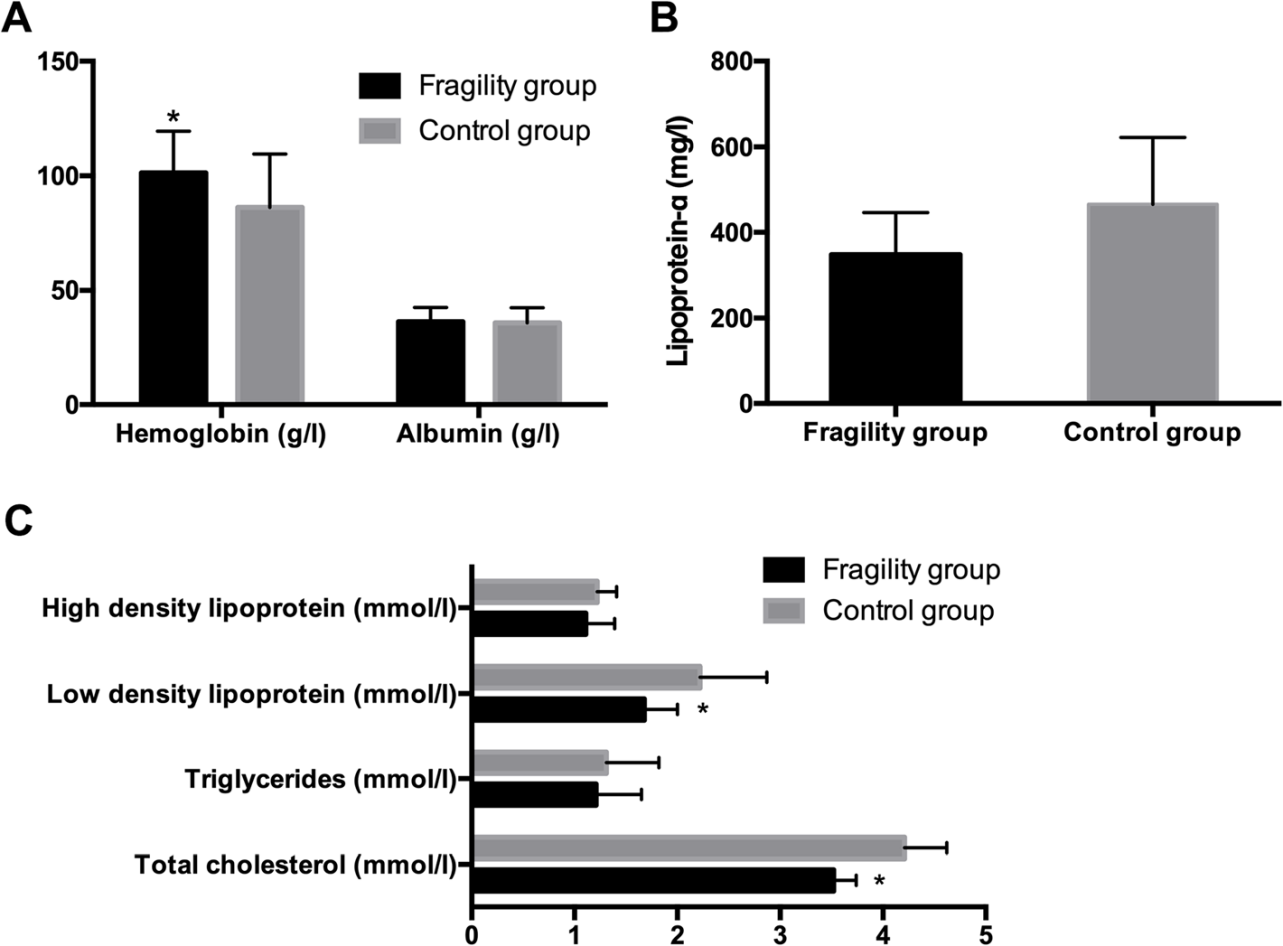
Nutrition-related parameters differences between FF and CG. **a**: Albumin and Hemoglobin in FF and CG, *: *p*< 0.05 when compared with CG. **b**: Lipoprotein-α in FF and CG, **c**: High density lipoprotein, Low density lipoprotein, triglycerides and Total cholesterol in FF and CG. *: *p*< 0.05 when compared with CG

**Fig. 2:**
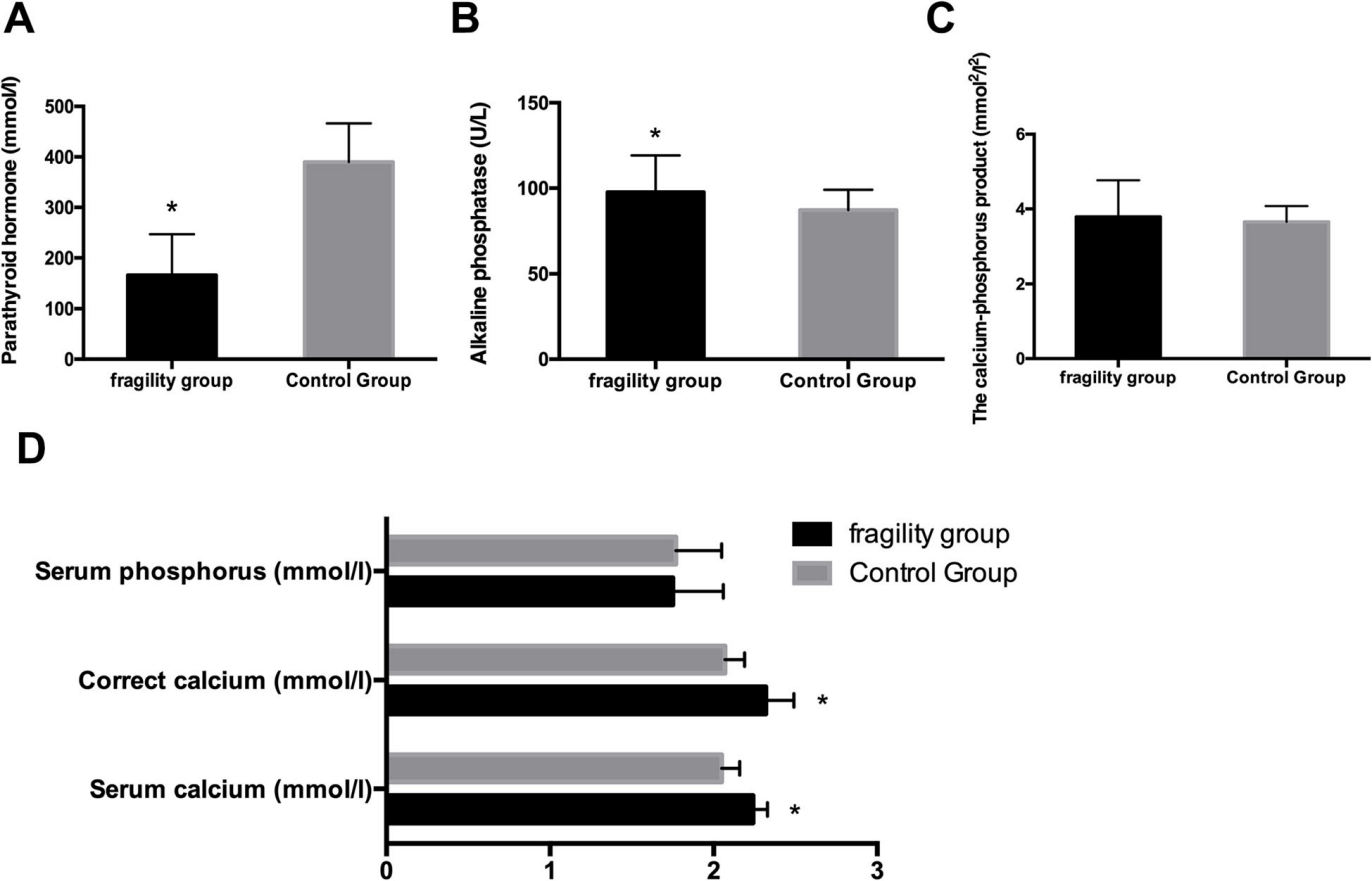
Mineral-bone metabolism disorders parameters between FF and CG. **a**: iPTH in FF and CG, *: *p*< 0.05 when compared with CG. **b**: Alkaline phosphatase in FF and CG, *: *p*< 0.05 when compared with CG. **c**: The calcium-phosphorus product in FF and CG. **d**: The serum phosphorus, correct calcium, serum calcium in FF and CG, *: *p*< 0.05 when compared with CG

In our study, the results showed that the FF patients had higher levels of hemoglobin than the CG patients (Fig. 1a). However, there were no statistically significant differences between the two groups in albumin (Fig. 1a), LP-α (Fig. 1b), TGs and HDL (Fig. 1c). What’s more, FF patients had lower levels of TC and LDL than the CG patients (Fig. 1c). The level of iPTH was lower in the FF group with statistical significance (Fig. 2a). However, it was also found that the serum levels of alkaline phosphatase (Fig. 2b), serum calcium and corrected calcium (Fig. 2d) were higher in the FF patients. There no significant difference in the calcium-phosphorus product (Fig. 2c) and serum phosphorus (Fig. 2d) between both groups.

### Analysis of risk factors for fragility fracture in ESRD patients

#### Logistic regression analysis of risk factors for fragility fracture in ESRD patients

We analyzed the variables whose *P* value was less than .1 by the logistic regression method. ESRD patients’ serum corrected calcium and alkaline phosphatase which acted as significant risks of fragility fractures were independent of the factors such as age, gender, and period of dialysis therapy. Normal levels of TC and iPTH might be protective against fractures. The results are shown in Table 2.

**Table 2:** Regression analysis results of risk factors for fragility fracture

	Wald Z	*P*	OR (95% CI)
Total cholesterol (mmol/l)	9.234	0.003	0.483 (0.301–0.768)
Alkaline phosphatase (U/L)	12.576	0.001	1.004 (0.989–1.010)
Correct calcium (mmol/l)	5.487	0.018	7.986 (2.402–46.897)
Parathyroid hormone (pg/ml)	9.905	0.002	0.912 (0.873–0.958)

(FF group: *n*=44; CG group: *n*=192)

#### Analysis of the relationship between different levels of bone metabolism parameters (serum calcium, serum phosphorus, and iPTH) and fragility fracture

**Fig. 3:**
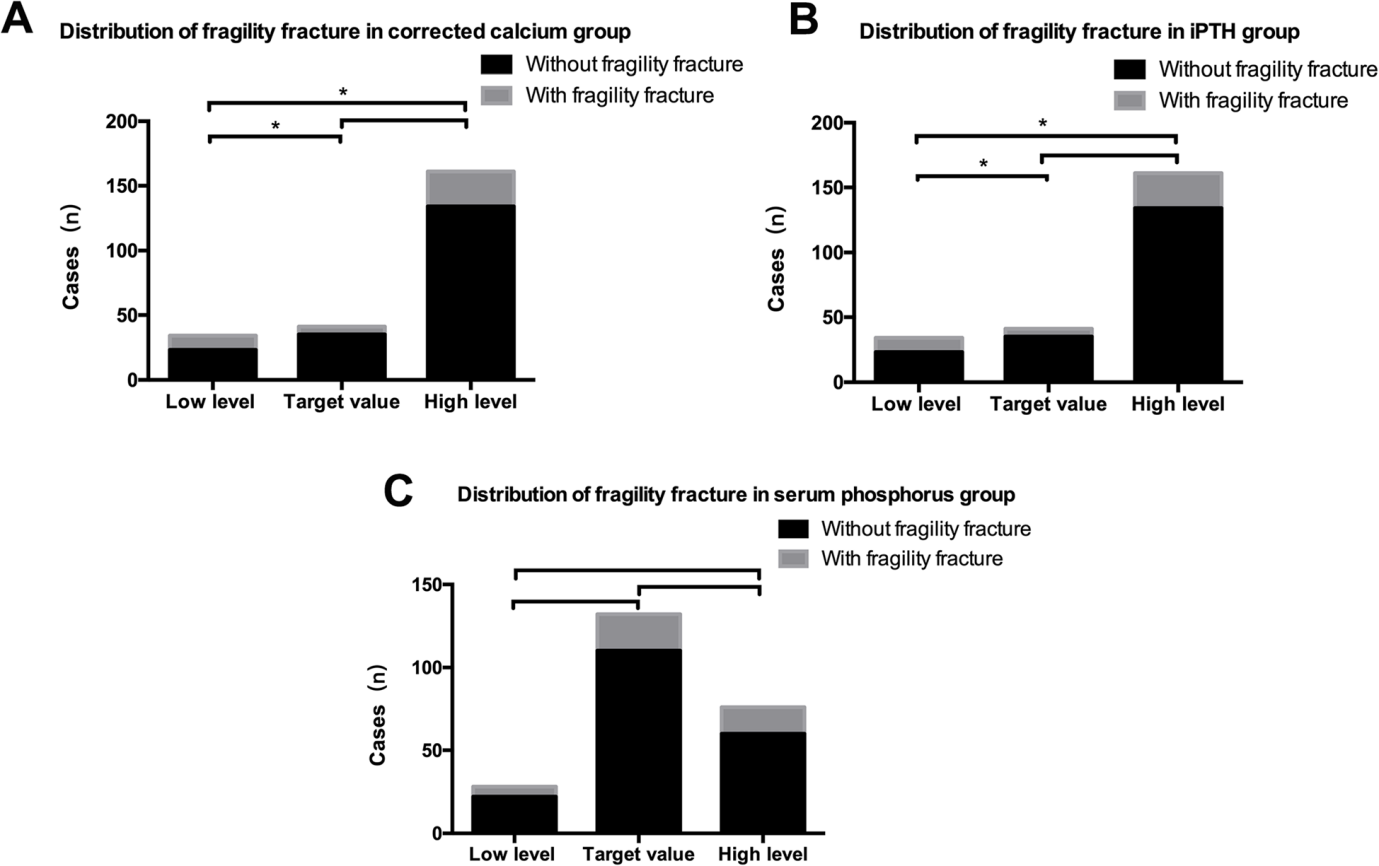
The distribution of fragility fracture cases in three subgroups of serum corrected calcium, phosphorus and iPTH. **a**. Distribution of fragility fracture in low level,target value and high level corrected calcium subgroup. *: *p*< 0.05 when the designated two subgroups were compared. **b**. Distribution of fragility fracture in low level,target value and high level iPTH subgroup. *: *p*< 0.05 when the designated two subgroups were compared. **c**. Distribution of fragility fracture in low level,target value and high level serum phosphorus subgroup. *: *p*< 0.05 when the designated two subgroups were compared

According to the KDIGO guidelines, the CKD5 patients’ therapeutic goal of correct calcium was 2.1–2.5 mmol/l and the goal was 1.13–1.78 mmol/l for serum phosphorus. We divided the patients into three different subgroups (low-level group, target value group and high-level group) according to the correct calcium and serum phosphorus therapeutic goals (supplementary Table 1). For iPTH, the therapeutic goal was 150–300 pg/ml according to the K/DOQI guidelines, and the patients were also divided into three subgroups as before. Analyzing fracture cases in each subgroup, the value group and the high-level group had a higher incidence of fragility fracture than the low-level group. At the same time, the low-level group of iPTH had a higher incidence of fragility fracture than the high-level and target value group. The differences were statistically significant (*P* < .05). All the results are shown in Fig. 3.

### Prognosis and survival analysis of ESRD patients suffering fragility fracture

The follow-up period of this study was 1476.0 (736.0, 2516.0) days. During the follow-up period, 12.1% of the patients were lost to follow-up and 79 died, 34 in the FF and 45 in the CG. Compared with the CG, the number of all-cause deaths and cardiovascular deaths in the FF showed a significantly higher incidence (*P* < .001) (Table 3). Eleven patients in the FF died from cardiovascular events, 13 patients died from septic shock, and 5 patients died from cachexia. Compared with the CG, the analysis results showed statistically significant differences.

**Table 3:** Distribution of death cause in all patients

	Fragility fracture group (*n*=44)	Control group (*n*=192)	*P* value
All-cause death	34 (77.3%)	45 (23.4%)	0.000
Cardiovascular event caused death	11 (25.0%)	16 (8.33%)	0.001
Myocardial infarction	2	2	
Cerebral apoplexy	5	3	
Congestive heart failure	1	1	
Sudden death	1	2	
Malignant arrhythmia	2	8	
Septic shock	13	8	0.001
Respiratory failure	5	13	NS
Cachexia	5	2	0.0005
Others (gastrointestinal hemorrhage, severe metabolic disorder. Etc.)	0	6	NS

**Fig. 4:**
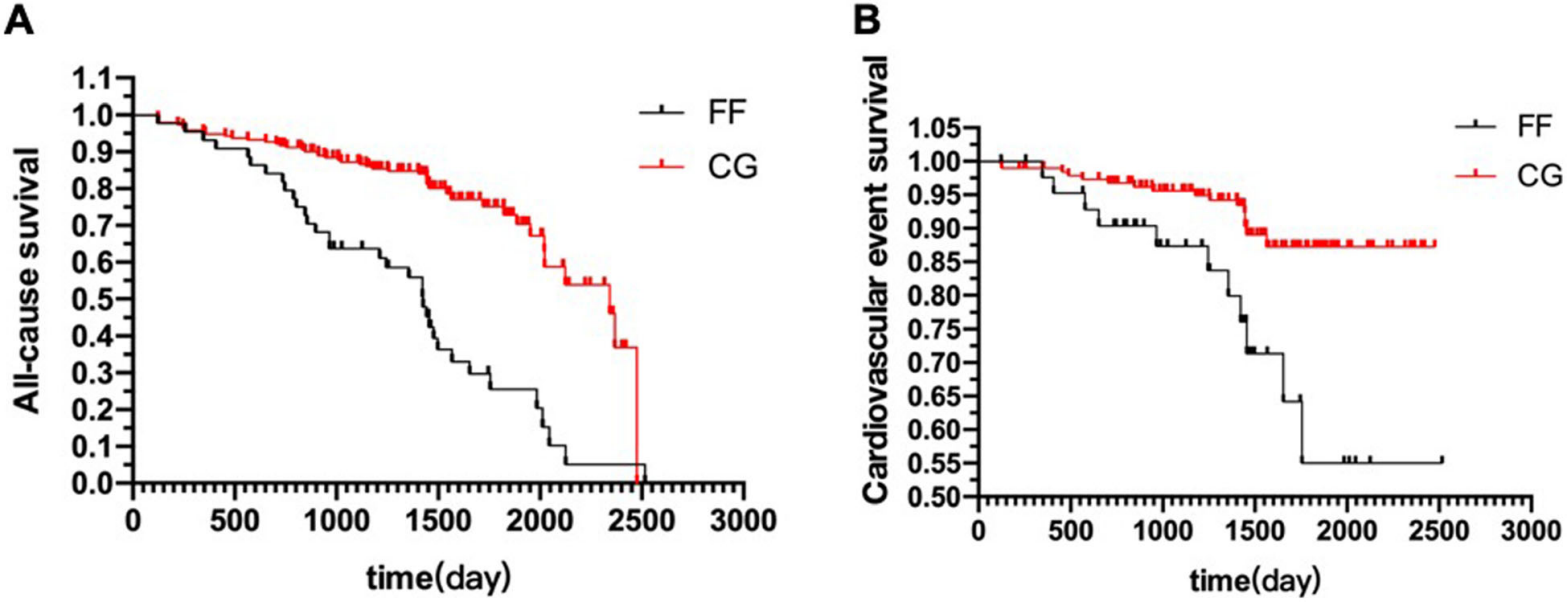
Kaplan-Meier survival curve analysis on the prognosis of ESRD patients. **a**. All-cause mortality of ESRD patients in the FF and CG group. **b**. Cardiovascular event survival curve in the FF and CG group

The prognosis of ESRD patients in the FF group was significantly poorer. Kaplan-Meier survival curve analysis showed that the all-cause mortality of ESRD patients in the FF group was significantly higher than that of the CG after 1 year of follow-up, and the survival rate decreased further after 5 years of uremia. The survival time of patients with fragility fracture was 246.0 (66.5, 758.0) days (Fig. 4a). Analysis of the cardiovascular event survival curve also showed that the ESRD patients in the FF group had significantly higher cardiovascular event mortality than the CG after 5 years of follow-up (Fig. 4b).

Further multivariate Cox regression analysis revealed that fragility fracture was an independent risk factor for all-cause mortality in ESRD patients (*P* < .001, RR: 4.877, 95% CI: 2.367–10.013). Other risk factors revealed include female gender and high levels of LDL (Table 4).

**Table 4:** Cox regression result for all-cause mortality

	Wald Z	*P*	OR (95% CI)
Female (0: no; 1: yes)	5.389	0.02	1.543 (1.076–2.179)
Low density lipoprotein (mmol/l)	5.265	0.019	1.256 (1.045–1.494)
Fragility fracture (0: no; 1: yes)	18.376	0.001	4.877 (2.367–10.013)

## Conclusion

1. Among dialysis patients with ESRD, the risk of fracture was greatly increased. The average annual incidence of fragility fracture in ESRD patients in this study was about 2.76‰. Proximal femoral fracture was the most common, followed by vertebral fractures.
2. Hypertension, diabetes, high levels of correct calcium, alkaline phosphatase, and excessive reduced iPTH level might be the risk factors of fragility fracture in patients with ESRD. Hypertension, correct calcium and alkaline phosphatase were independent risk factors. Serum nutrition-related parameters, and appropriate levels of parathyroid hormone might be protective factors.
3. The prognosis of ESRD patients in the FF group was significantly poorer. Fragility fracture was an independent predictor of all-cause death in patients with ESRD. Cardiovascular event, along with septic shock, become the leading causes of death in ESRD patients with fragility fracture.

## Discussion

Chronic kidney disease (CKD) is common, with an estimated worldwide prevalence of 7% of patients in stage 3–5. In addition, with the aging of society, the incidence of chronic kidney disease, especially ESRD, is gradually increasing [6]. Due to the advanced stage of chronic renal disease, renal osteodystrophy often occurs in ESRD patients, and subsequent fractures are common. Renal osteodystrophy usually interferes with bone metabolism and related hormone levels to reduce bone mass, strength and cause abnormal bone remodeling [7, 8]. These bone abnormalities are common in most ESRD patients, especially those requiring dialysis [9]. Therefore, patients with ESRD have a higher risk of fracture, and the risk of fracture increases as renal function declines. Fragility fractures in ESRD patients are a serious complication, resulting in high morbidity, high mortality [10], increased financial burden and prolonged hospitalization [11, 12]. In recent years, there have been relatively few studies on fragility fractures in ESRD patients, and most have focused on cases from Europe and North America. Whether the results can be extrapolated to Asian populations is unclear because of differences in race and social structure. In our study, Chinese patients were the main subjects. Among the 521 patients studied, we found that the incidence of fragility fractures during the follow-up period was 8.4%, proximal femoral fractures and vertebral body fractures were the main types seen. The annual incidence of fragility fractures during follow-up was 2.76‰. Compared with other research, the incidence of fractures was similar, although our study showed an obvious decrease in annual incidence compared with data from the research of Tseng et al. [13]. This may be related to our workload, which has over 2600 hemodialysis treatments per week. Dialysis patients are treated with high quality normative therapy in our hospital. The dialysis center nursing department has excellent education on the causes of fracture such as falls and dizziness.

Our study found that the FF group patients were significantly more likely to have underlying diseases such as essential hypertension and diabetes than the CG, and our subsequent correlation analysis showed that essential hypertension was an independent risk factor for fragility fractures. This conclusion is consistent with that reported by Yang, Sennerby, and Vestergaard P et al. [14–16]. We suspect that the reason may be linked to the high incidence of symptoms such as dizziness which occurs with blood pressure fluctuation, and high blood pressure-related urinary calcium loss which can cause bone quality decline. Although our experiment did not investigate this further, a literature search revealed that Rull [17] recommended a 24-h urinary calcium and empty stomach calcium/creatinine ratio test that could offer better data support. This can be considered in future studies. We also hypothesize that diabetes causes blood glucose fluctuations that may result in symptoms such as dizziness, amaurosis fugax, or nausea and thus increase the risk of falls leading to fractures. Existing research [18–20] has detailed the association between diabetes and osteoporosis. Other results of studies into osteoporosis and fragility fractures are consistent with our research.

In this study, the nutritional indicators including hemoglobin, serum TC and LDL in patients with fragility fractures were significantly lower than those in the CG, while the levels of TG, high-density lipoprotein and apolipoprotein were slightly lower than those in the CG, but there was no statistical significance. This situation suggests a relationship between fragility fractures and nutritional status. The logistic regression analysis in this study suggests higher TC levels may be a protective factor for fractures. Sivas et al. [21] believe that the increase of TC could reduce the risk of vertebral fracture proportionately. Yamaguchi et al. [22] suggested that an increase of one standard deviation of TG reduced the risk of vertebral fracture in perimenopausal women by approximately 50%. However, Wang Y et al. pointed out that although high levels of TG were associated with fragility fractures, there was no relationship between increased TC at standard deviation and fracture susceptibility [23]. The study by Trimpou et al. [24] found that high TC was directly associated with fracture susceptibility. We believe that the conflict between the two results achieved from the healthy people and ESRD patients may be the result of significant confounding factors. The relevant serum lipids and bone metabolism which are directly affected by chronic kidney disease and the common effect of statins and double phosphate on bone and lipid metabolism may have introduced biases. We believe that a moderately high level of cholesterol often represents a better nutritional status and is associated with effective dialysis. These patients have a better quality of life and are active so they have better motor strength and coordination of their skeletal muscles, thus reducing the risk of fragility fracture. We recommend a follow-up study with an expanded sample size in ESRD patients to confirm our speculation.

We found that ESRD patients in the FF group had higher serum calcium, serum corrected calcium and alkaline phosphatase levels than the CG, while iPTH levels were lower than the CG. Similarly, in the comparative study based on the levels recommended by KDIGO, the fracture rate of the target level and the high corrected calcium group was higher than that of the low corrected calcium group, and the fracture rate of the low iPTH group was higher than that of the target level and the high iPTH group. The regression analysis also confirmed that fragility fracture was correlated with the low iPTH and a high level of corrected calcium. We speculate that this might be related to low calcium and high phosphatemia in CKD patients, resulting in prescribed calcium supplements or calcitriol. High alkaline phosphatase and high serum calcium not only did not lead to increased bone calcium deposition but also led to excessive inhibition of iPTH and failure of osteoblastic transformation and function. These processes affected the mineralization of bone and led to increased fracture susceptibility. The relationship between low iPTH and fracture has been confirmed in the studies of Matias PJ [25] and Atsumi [26]. The study of Maruyama et al. [27] also confirmed that elevated alkaline phosphatase in ESRD patients increased the risk of fracture. In many of the reports we reviewed, the significant effects of hyperphosphatemia on the prognosis of ESRD patients has been repeatedly noted, but in our study, there was no significant difference in serum phosphorus levels between the fracture group and the CG, and further studies have shown no significant difference in fracture incidence among the subgroups of serum phosphorus defined by KDIGO. This may reveal that although hyperphosphatemia is closely related to the prognosis of ESRD patients, it is not a risk factor for brittle fracture. The serum level of vitamin D3 was not included in this study, which may be a confounding factor. Further research will be carried out in follow-up experiments.

In this study, we found that 77.3% of the patients died in the FF group and 25.0% of them died from the cardiovascular event. Both all-cause mortality and the cardiovascular event mortality were higher in the FF group. it was found that the survival rate and life span of patients with fragility fractures decreased significantly. We speculate that the impact of fragility fractures on the risk of death is due firstly, to an increased risk of cardiovascular events. Shantouf et al. [28] showed that high serum alkaline phosphatase may be associated with metastatic calcification of soft tissue. Therefore, high levels of alkaline phosphatase in ESRD patients with fragility fractures may promote arterial and cardiac calcification. In our study, the COX regression analysis showed that high LDL was an independent risk factor for all-cause mortality, and high LDL was also a risk factor for arterial calcification and cardiac valve calcification [29]. There are limitations in this study. Firstly, based on the exclusion criteria, there were potential selection bias between the 236 study population and the excluded patients. The case: control ratio was more than 1:4 which may not improve statistical power and not all of the study population had X-ray and CT scan which may cause potential selection bias. Limited by the recording system, the drug mineral metabolism related therapies and the dialysis characteristics were not present in detail. Secondly, this study did not confirm the calcification of artery and heart valve in patients, this should be investigated in future studies. Thirdly, there may be an increased risk of infection. Fractures can lead to prolonged bed rest, an increased risk of hypostatic pneumonia, and a risk of developing refractory systemic infection, leading to septic shock and systemic multi-organ failure. We found that the fracture group had more deaths from septic shock and multiple organ dysfunction than the CG, which supports our hypothesis. Studies by Groff [30], Dodd [31] and many scholars have also confirmed the association between secondary infection after fracture and patient death.

## Supplementary Information

**Additional file 1: Supplementary Table 1**. The distribution of fragility fracture cases in three subgroups of serum corrected calcium, phosphorus and iPTH.

## Data Availability

The datasets analysed during the current study are not publicly available due to the potential use in further researches but are available from the corresponding author on reasonable request.

## Abbreviations

ACEIs: Angiotensin-converting enzyme inhibitors
ARB: Angiotensin receptor blockers
CG: Control group
CKD: Chronic kidney disease
ESRD: End-stage renal disease
FF: Fragility fracture
iPTH: Immunoreactive parathyroid hormone
KDIGO: Kidney disease improving global outcome
LDL: Low density lipoprotein
rh EPO: Recombinant human erythropoietin
TC: Total cholesterol
