# Supplementary material for: A retrospective study of end-stage kidney disease patients on maintenance hemodialysis with renal osteodystrophy-associated fragility fractures
Source: BMC Nephrol. 2021 Jan 11;22:23. doi: 10.1186/s12882-020-02224-7 (PMC7802139; doi:10.1186/s12882-020-02224-7)
Supplement: Supplementary file 1 — Additional file 1: Supplementary Table 1. The distribution of fragility fracture cases in three subgroups of serum corrected calcium, phosphorus and iPTH. [file 12882_2020_2224_MOESM1_ESM.docx]

Supplementary table 1 The distribution of fragility fracture cases in three subgroups of serum corrected calcium, phosphorus and iPTH

| Parameters | Group | Number of patients (n) | | Without fragility fracture (n) | With fragility fracture (n) |
| --- | --- | --- | --- | --- | --- |
| corrected calcium | Low level | 92 | | 85 | 7 |
|  | Target value | 133 | | 98 | 35 |
|  | High level | 11 | 9 | | 2 |
| serum phosphorus | Low level | 28 | 22 | | 6 |
|  | Target value | 132 | 110 | | 22 |
|  | High level | 76 | 60 | | 16 |
| iPTH | Low level | 34 | 23 | | 11 |
|  | Target value | 41 | 35 | | 6 |
|  | High level | 161 | 134 | | 27 |

iPTH: immunoreactive parathyroid hormone.
